# Trends in complementary/alternative medicine use by breast cancer survivors: Comparing survey data from 1998 and 2005

**DOI:** 10.1186/1472-6874-7-4

**Published:** 2007-03-30

**Authors:** Heather S Boon, Folashade Olatunde, Suzanna M Zick

**Affiliations:** 1 Leslie Dan Faculty of Pharmacy and Department of Pharmaceutical Sciences, University of Toronto, 144 College Street, Toronto Ontario, M5S 3M2, Canada; 2 Department of Pharmaceutical Sciences, University of Toronto, 144 College Street, Toronto Ontario, M5S 3M2, Canada; 3 University of Michigan Comprehensive Cancer Center and University of Michigan Department of Family Medicine, 715 E. Huron, Suite 2E, Ann Arbor, MI 48104, USA

## Abstract

**Background:**

Use of complementary and alternative medicine (CAM) by women with breast cancer is often said to be increasing, yet few data exist to confirm this commonly held belief.

The purpose of this paper is to compare overall patterns of CAM use, as well as use of specific products and therapies at two different points in time (1998 vs 2005) by women diagnosed with breast cancer.

**Methods:**

Surveys were mailed to women randomly selected from the Ontario Cancer Registry (Canada) in the spring of 1998 (n = 557) and again in the spring of 2005(n = 877).

**Results:**

The response rates were 76.3% in 1998 and 63% in 2005. In 1998, 66.7% of women reported using either a CAM product/therapy or seeing a CAM therapist at some time in their lives as compared with 81.9% in 2005 (p = 0.0002). Increases were seen in both use of CAM products/therapies (62% in 1998 vs. 70.6% in 2005) and visits to CAM practitioners (39.4% of respondents in 1998 vs 57.4% of respondents in 2005). Women in 2005 reported that 41% used CAM for treating their breast cancer. The most commonly used products and practitioners for treating breast cancer as reported in 2005 were green tea, vitamin E, flaxseed, vitamin C, massage therapists and dietitians/nutritionists.

**Conclusion:**

CAM use (both self-medication with products and visits to CAM practitioners) increased significantly from 1998 to 2005. Now that more than 80% of all women with breast cancer report using CAM (41% in a specific attempt to management their breast cancer), CAM use can no longer be regarded as an "alternative" or unusual approach to managing breast cancer.

## Background

The importance of breast cancer in women, as a public health problem, is well recognized worldwide [1,2]. In Canadian women, breast cancer is a common malignant neoplasm, with a lifetime risk calculated as 1 in 9 [3]. Due to mammographic screening, early detection of disease, and improved therapies, the rate of BC mortality is steadily declining in Canada [3]. Nonetheless, in 2005, it is estimated that 21,600 women were diagnosed and 5,300 women died from this disease [3]. Ontario has the highest incidence of BC cases in Canada, with 8,200 of the 21,600 new cases in 2005 [3].

There have been many surveys of CAM use among patients with breast cancer in the US, Britain, and Canada. Survey results among breast cancer patients find that prevalence of CAM use ranges from 16.5 percent to 84 percent [4-9]. Our own study of a random sample of breast cancer survivors in Ontario, Canada found that 66.7 percent had used some form of CAM [8]. Women cite numerous reasons for taking alternative treatments including to reduce physical distress, fatigue, anxiety, depression, sleep disturbance, nausea, weight change, hair loss, diminished concentration, and pain.

Many reports suggest that the prevalence of CAM in cancer patients and survivors has been increasing, yet few follow-up studies are available to confirm this. In addition, the use of different definitions of CAM, and thus different instruments to measure CAM prevalence, makes it difficult to compare studies completed at different points-in-time. The primary purpose of this paper is to compare overall patterns of prevalence of CAM use, as well as use of specific CAM products and therapies in two random samples of women diagnosed with breast cancer in 1998 and seven years later in 2005, using the same survey questions and definition of CAM. Specifically, CAM was defined as medical interventions that are not taught widely in medical schools or generally available in hospitals[10] In addition, the clinical, sociodemographic, and characteristics of CAM consumers in 1998 are compared with those of consumers in 2005.

## Methods

**Figure 1:**
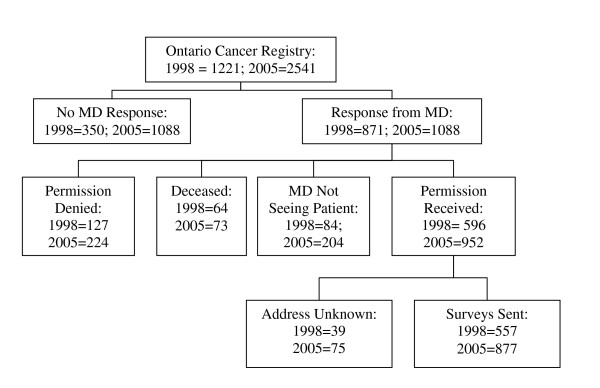
Derivation of the sample.

At both time points (1998 and 2005), a random sample of women 18 years and older and diagnosed with breast cancer were selected from the Ontario Cancer Registry based on pathology reports (See Figure 1). A mailed reminder letter was posted approximately one week following the initial mail out. In addition, approximately three weeks after the initial mailing, a final reminder letter and second survey was mailed to each non-respondent. This Registry is a computerized database that contains information on virtually all Ontario residents diagnosed with cancer. As stipulated by the Registry, permission to contact each woman was requested from her family physician or oncologist. Up to three telephone reminders were provided in attempts to obtain responses from as many physicians as possible. Surveys were mailed to all women for whom permission and mailing addresses were received. Women who did not respond to two follow-up letters were identified as non-responders. See Figure 1 for details on the derivation of the sample.

The questionnaire asks women to identify which CAM practitioners and CAM therapies/products (from a specific list) they had ever visited or used. In 2005, each respondent was also asked to indicate if she visited the practitioners and/or used the therapies for her breast cancer or for some other reason. In both years, demographic information and details about each participant's cancer (e.g., stage, conventional treatment, hormone receptor status) were also collected.

### Statistical analysis

Respondents of the 2005 survey were categorized as CAM consumers or nonconsumers. CAM consumers were further categorized as either using or not using particular CAM products or visiting CAM practitioners. We calculated frequencies, counts, and means to describe the pattern of clinical, sociodemographic and prevalence of CAM practitioners and products characteristics for respondents of the 2005 survey. To compare the relationship of respondents of the 2005 group who were CAM consumers to nonconsumers on sociodemographic and clinical characteristics we calculated Fisher Exact tests for categorical variables and independent *t*-tests for continuous variables. We calculated Z-values for independent proportions (two-sided) to compare sociodemographic and clinical characteristics as well as the prevalence of CAM products used and practitioners visited between the 1998 and the 2005 respondents. The data were analyzed using Statistical Package for Social Sciences for Windows, version 12.0 (SPSS, Chicago, ILL).

This study was approved by the University of Toronto Ethics Review Office and the Institutional Review Board for Human Subject Research at the University of Michigan Medical School.

## Results

In 1998, the survey was mailed to 557 women and the final response rate was 76.3 percent (411/539). Seven of the 1998 respondents were reported to have died and 11 women reported that their first diagnosis of cancer was prior to 1994 and thus did not meet the inclusion criteria for the study. Thus these women were not counted in the denominator when calculating the response rate for the study. See Boon et al. 2000 for additional details on the 1998 data collection [8]. In 2005, the survey was mailed to 877 women who were diagnosed with breast cancer between 2001 and 2003. A total of 541 surveys were returned and sixteen families provided information that the potential participant had died. In addition, 31 surveys were removed from the analysis because the individual reported her date of diagnosis as being prior to 2001. Thus the final response rate in 2005 was 63 percent (541/(877-16)). See Zick et al. 2006 for additional details on the 2005 data collection[11]

### Participants

Table 1 provides demographic and breast cancer diagnosis/treatment data. The 2005 group was surveyed significantly closer to their date of diagnosis compared to the 1998 group and had a slightly higher level of education. Compared to the 1998 cohort, significantly fewer respondents in 2005 reported having surgery, but significantly more reported having chemotherapy and hormone therapy for their breast cancer.

**Table 1:** Patient characteristics

Characteristics	1998 N = 411 *n *(%) or Mean (SD)	2005 N = 527 *n *(%) or Mean (SD)	P-value*
Mean age (years)	57.9 (11.3)	60.9 (13.3)	<0.01^(1)^
Months since first diagnosis with BC†§	34.9 (8.75)	27.5 (5.4)	<0.01^(1)^
Had surgery for their BC	394 (95.8)	479 (90.8)	<0.01^(2)^
Had radiotherapy for their BC	263 (64.1)	350 (66.5)	0.44^(2)^
Had chemotherapy for their BC	152 (37.1)	234 (44.4)	0.02^(2)^
Had hormone therapy for their BC	114 (27.8)	273 (51.8)	<0.01^(2)^
Married	294 (71.6)	335 (63.5)	0.01^(2)^
North American/European cultural group	337 (82.0)	405 (76.8)	<0.01^(2)^
Completed more than high school	202 (49.2)	295 (56.0)	0.03^(2)^
Household annual income > $40,000§	204 (49.7)	245 (46.4)	0.54^(2)^

* (1) Based on an independent sample t-test, (2) based on a two-sided Z-score

† Calculated as the number of months between a woman's date of diagnosis to April 1^st ^1998 for the 1998 cohort and to September 1^st ^2005 for the 2005 cohort

± BC= Breast cancer

§Income based on Canadian dollars for total household income (not specified if before or after taxes) and total household income in Canadian dollars before taxes in 2005 cohort

### Use of CAM

In 2005, 81.9 percent of women reported using either a CAM product/practice or seeing a CAM therapist at some time in their lives as compared with 66.7 percent in 1998 (p = 0.0002). Increases were seen in both use of at least one CAM product or therapy (70.6 percent in 2005 vs. 62 percent in 1998) and visits to CAM practitioners (57.4 percent of respondents in 2005 versus 39.4 percent or respondents in 1998). In both years, the median number of CAM practitioners ever seen was one and the median number of products/practices ever used was two. Women in 2005 reported that 41 percent (n = 220) used CAM as part of the management of their breast cancer. The most commonly used products and practitioners for managing breast cancer included green tea, vitamin E, flaxseed and vitamin C, massage therapists and dietitians/nutritionists (see Table 2). Questions about the use of CAM specifically to manage breast cancer were not asked in the 1998 survey.

**Table 2:** CAM Products and Practitioners most commonly used by 2005 cohort for symptoms associated with their breast cancer

**Product**	***n *(%)**
Green Tea	71 (13.3)
Vitamin E	70 (13.2)
Flax Seeds	66 (12.4)
Vitamin C	65 (12.3)
Special Food/Diets	53 (10.0)
Meditation	39 (7.4)
Fish Oil	35 (6.6)
Beta-carotene	32 (6.0)
Essiac	29 (5.5)
Other*	28 (5.3)
Soy	27 (5.1)
	
**Practitioner**	***n *(%)**
Massage Therapist	51 (9.8)
Dietitian/Nutritionist	45 (8.5)
Reiki Practitioner	26 (4.9)
Naturopath	25 (4.7)
Other*	24 (4.5)
Homeopath	23 (4.3)
Therapeutic Touch Practitioner	19 (3.6)
Herbalist	14 (2.6)
Reflexologist	12 (2.3)
TCM Practitioner†	12 (2.2)
Chiropractor	11 (2.1)

* Other products include yoga, chelation therapy, music/art therapy, noni juice/rain tree products, visualization, relaxation therapy, aboriginal medicine, exercise, flower essence, progesterone and other practitioners include aboriginal sweat & healing, brain respiration, cancer consultant, doctor of biomolecular medicine, exercise, iridologist, lymph clinic, muscle therapy, music and art therapy, occupational/physiotherapist, osteopath,

† TCM = Traditional Chinese Medicine

Table 3 provides details on use of specific CAM practitioners and products in 1998 compared to in 2005. Significant increases were seen in use of body work practitioners (including Reiki practitioners, massage therapists, therapeutic touch practitioners and shiatsu practitioners), acupuncturists/traditional Chinese medicine (TCM) practitioners, homeopathic practitioners and a general "other" category in 2005 compared to 1998. Significant increases were also seen in use of herbal remedies overall and specifically in the use of green tea and special foods/diets. Use of Essiac (Essiac is a blend of at least four herbs (burdock root (*Arctium lappa*), Indian rhubarb (*Rheum palmatum*), sheep sorrel (*Rumex acetosella*) and the inner bark of slippery elm (*Ulmus fulva *or *U. rubra*) significantly decreased.

**Table 3:** Use of CAM: 1998 *versus *2005

	**Respondents Reporting CAM** **Use** ***n *(%)**		P-value*
**Visits to Practitioners**	**1998** **N = 411**	**2005** **N = 527**	
Chiropractor	120 (29.2)	180 (34.2)	0.11
Body work practitioners†	94 (22.9)	228 (43.3)	>0.01
Acupuncturist/TCM practitioner±	28 (6.8)	69 (13.0)	>0.01
Herbalist	28 (6.8)	25 (4.7)	0.17
Naturopathic practitioner	24 (5.8)	40 (7.6)	0.29
Reflexologist	24 (5.8)	46 (8.7)	0.09
Homeopathic practitioner	17 (4.1)	41 (7.7)	0.02
Physician offering CAM therapies±	17 (4.1)	N/A	N/A
Spiritual/faith healer	16 (3.9)	N/A	N/A
Other visits^(1)^§	7 (1.7)	36 (6.8)	>0.01
Colon irrigation	4 (1.0)	N/A	N/A
Ayurvedic practitioner	N/A	4 (0.8)	N/A
Craniosacral therapist	N/A	10 (1.9)	N/A
Dietician/Nutritionist	N/A	104 (19.7)	N/A
			
**Use of Products**	**1998** **N = 411**	**2005** **N = 527**	
Vitamins/Minerals^(1) ^#	204 (49.6)	269 (51.0)‡	0.67
Herbal Remedies^(2) ^#	101 (24.6)	194 (36.8)*	>0.01
Green tea	71 (17.3)	145 (27.5)	>0.01
Special foods/diet	63 (15.3)	140 (26.6)	>0.01
Essiac	61 (14.8)	39 (7.4)	>0.01
Meditation	42 (10.2)	60 (11.4)	0.57
Shark cartilage	22 (5.4)	16 (3.0)	0.07
Other therapies^(2) ^§	22 (5.4)	39 (7.4)	0.21
Homeopathy	16 (3.9)	34 (6.4)	0.08
Faith healing	14 (3.4)	N/A	N/A
TCM remedy	7 (1.7)	18 (3.4)	0.11
Natural Supplements^(3) ^#	N/A	198 (37.6)†	N/A
Soy	N/A	58 (11.0)	N/A

* Z-value for two independent proportions (two-sided) looking at change in product or practitioner use through time

†Body Work includes: reiki practitioner, massage therapist, therapeutic touch therapist, shiatsu practitioner, and pranic healer

± TCM = Traditional Chinese Medicine, CAM = complementary and alternative medicine

§(1)Others visits include: aboriginal sweat & healing, brain respiration, cancer consultant, doctor of biomolecular medicine, exercise, iridologist, lymph clinic, muscle therapy, music and art therapy, occupational/physiotherapist, osteopath, (2) Glucosamine/chondrotin, yoga, chelation therapy, music/art therapy, noni juice/rain tree products, visualization, relaxation therapy, aboriginal medicine, exercise, flower essence, progesterone

# (1) Vitamins/minerals (other than a multi-vitamin) include beta carotene and vitamins C and E; (2) Herbal remedies include garlic, ginger, and Hoxsey formula (or tea), Essiac, aloe, ginseng, green tea/extract : and (3) Natural supplements include acidophilus (or probiotics), CoQ_10_, fish oil, flax seed, immune support (e.g. MGN_3 _or Coriolus) and melatonin

## Discussion

Overall, we found that both the use of CAM products and visits to CAM practitioners by women diagnosed with breast cancer significantly increased from 1998 to 2005. In 2005, 81.9 percent of respondents reported using CAM (41 percent to help manage their breast cancer) compared to 66.7 percent in 1998, suggesting that in 2005 CAM use has become the "norm" in this patient population.

The observed increase in CAM use appears not to be explained by changing demographics in the breast cancer population. The two cohorts were approximately the same with respect to age and income levels. The differences in ethnicity, the percentage that were married, and the level of education appear not to be big enough to account for the increases in use. For example, a mean age difference of 3 years is unlikely to be clinically significant. The 2005 cohort reported less surgery, but more chemotherapy and more hormone therapy (e.g., tamoxifen) which may be a reflection of differences in stage of cancer at diagnoses; however since cancer stage information was not collected in 1998, it is not possible to assess this. The differences in conventional treatments may also be related to changes in therapeutic protocols for breast cancer that have occurred since 1998.

Overall, the biggest increases were seen in the percentage of women seeing bodywork practitioners (including massage therapists, but not chiropractors), TCM practitioners/acupuncturists, homeopaths and "others". This may be related to the fact that massage therapy and acupuncture are two of the CAM practices which are generally the most accepted by the medical profession [12-15]. For example, many physicians use acupuncture in their practices [16]. In addition, given that neither acupuncture nor massage involves taking anything orally, it is likely they are perceived to have fewer potential adverse effects or interactions with conventional cancer treatments than some other CAM therapies. So it is possible that conventional MDs are either recommending these options more often to patients or at least not discouraging patients from using them.

Perception of safety and perceived lack of drug interactions may also explain the increased use of homeopathy. In addition, the homeopathic community was under review by the Health Professions Regulation Advisory Council for consideration as a future regulated health profession in Ontario during 2005 when this survey was conducted [17]. Although the council hearing did not receive much media attention at the time, it may partially explain the increased use of homeopathy reported here.

Overall, there was a significant increase in the use of herbal products such as garlic, ginger, ginseng and green tea and special foods/diets. Both green tea and special diets/foods are used by more than 10 percent of all women diagnosed with breast cancer specifically to help manage their breast cancer. A review of the evidence suggests that there may be an overall decreased risk of cancer associated with taking Asian ginseng (*Panax ginseng*), garlic, green tea, soy, and tomatoes but research is extremely preliminary and in some cases contradictory. Also, the minimum dose associated with decreased cancer risk has not been clearly defined for any of the supplements or herbs [18,19]. Although there is evidence that ginger may be useful in the management of chemotherapy-induced nausea, it is not clear exactly what dose or dosing schedule is best. It is also not clear what additional benefits ginger products have over and above conventional anti-nausea medications (fewer adverse effects are claimed) [18,19]. There are currently no herbs with significant evidence of efficacy as cancer treatments [18,19].

Our results also found the use of Essiac significantly decreased. Essiac has been used in Canada for over 70 years [20]. In contrast to green tea and soy, which has received considerable media and scientific attention as possible cancer prevention agents, Essiac has received little notice and has no peer-reviewed clinical trials or encouraging animal studies demonstrating any significant beneficial effects. For example, our retrospective survey found that Essiac did not have a significant effect on either health related quality of life or mood states in women with breast cancer. Women in the study were taking low doses (total daily dose 43.6 ± 30.8 mL) of Essiac that corresponded to label directions found on most Essiac products, but appear unlikely to have significant pharmacological effects[11] Thus, it appears at least in part, that women are using more natural health products that are receiving increased positive attention in the media and by the scientific community and decreasing those that have garnered little evidence or interest.

Several limitations of our study need to be addressed. As with any retrospective study recall bias is a possible limitation. In 1998, respondents were only asked if they had ever used CAM products or visited CAM therapies. Thus, although we can comment on what women in 2005 were using to help manage their breast cancer, it is not possible to compare this to women in 1998.

Both in 1998, and in 2005, the most up-to-date records from the Ontario Cancer Registry were used to select samples as close to diagnosis as possible. However, given the recent computerization of the Registry, the 2005 cohort was surveyed almost 12 months closer to their diagnosis of cancer than the 1998 cohort and thus may have been more active in using CAM products and therapies to manage the lingering side effects of conventional cancer treatments. It might also have been easier for these women to recall the CAM they had used in the more active symptom phase of the management of the cancer than the women surveyed in 1998. It is possible that the difference in time since diagnosis accounts for the changes in CAM use reported by the two cohorts.

We originally selected a random sample from the Ontario Cancer Registry; however we were only able to mail surveys to approximately one-half of the sample from 1998 and just over one-third of the sample in 2005 (see Figure 1). Physicians declined to provide permission for women that were too ill or emotionally unstable to respond or were unable to speak English (less than ten percent of the original sample). The reason we were unable to contact most of the other women was our inability to obtain a response from the physicians we contacted to obtain permission to mail a survey to the patient. It is possible that physicians who did not respond had more negative attitudes toward CAM and may have influenced their patients not to take CAM than those who agreed to allow their patients to participate. Thus, our prevalence rates may be an over-estimate of the actual prevalence of CAM use in the population of women with breast cancer.

## Conclusion

Our findings suggest that the majority of women with breast cancer use CAM in combination with conventional medical breast cancer treatments and this highlights the importance of additional research on the safety, efficacy and interactions of these products and therapies. It is important for clinicians to routinely ask patients about their CAM use, but making recommendations about use, especially in conjunction with chemotherapy, radiation therapy and hormone therapy remains problematic due to lack of research of the potential benefits or harmful effects of these combinations.

CAM use (both self-medication with products and visits to CAM practitioners) increased from 1998 to 2005 in our samples. Now that more than 80 percent of all women with breast cancer report using CAM (41 percent in a specific attempt to management their breast cancer), CAM use can no longer be regarded as an "alternative" or unusual approach to managing breast cancer. The increasing popularity of CAM increases the urgency for research into the safety and efficacy of these products and therapies.

## Abbreviations

CAM – complementary and alternative medicine

TCM – traditional Chinese medicine

## Competing interests

The author(s) declare that they have no competing interests.

## Authors' contributions

HB and SZ conceived and designed the study. HB organized and supervised data collection and inputting; drafted the paper and approved the final version. SZ organized and supervised the data analysis, provided critical comments on various drafts of the paper and approved the final version. SO helped to develop and pre-test the survey instrument, helped to organize data collection, commented on drafts of the paper and approved the final version. All authors read and approved the final manuscript.

## Pre-publication history

The pre-publication history for this paper can be accessed here:


